# Precision Antisense Oligonucleotide Therapy Amenability for Infantile Genetic Epilepsies

**DOI:** 10.1001/jamaneurol.2026.1021

**Published:** 2026-05-04

**Authors:** Emma Sherrill, David Cheerie, Cara J. Beck, Ella F. Whittle, Yasin Shafi, Natalie J. Chandler, John Christodoulou, Jerusalem Daniel, Jane Hassell, Maria Lachgar-Ruiz, Sarah Mulhern, Elizabeth Scotchman, Jashanpreet Sidhu, Celine Florentia Tedja, Lyn S. Chitty, J. Helen Cross, Ingrid E. Scheffer, Haiyan Zhou, Timothy W. Yu, Vann Chau, Sarah E. M. Stephenson, Annapurna Poduri, Katherine B. Howell, Amy McTague, Gregory Costain, Alissa M. D’Gama

**Affiliations:** 1 Division of Genetics and Genomics, Department of Pediatrics, Boston Children’s Hospital, Boston, Massachusetts; 2 Program in Genetics and Genome Biology, SickKids Research Institute, Toronto, Ontario, Canada; 3 Murdoch Children’s Research Institute, Parkville, Victoria, Australia; 4 Genetics and Genomic Medicine, UCL Great Ormond Street Institute of Child Health, London WC1N 1EH, United Kingdom; 5 UK Platform for Nucleic Acid Therapies (UPNAT), London, United Kingdom; 6 North Thames Genomic Laboratory Hub, Great Ormond Street Hospital NHS Foundation Trust, London, United Kingdom; 7 Department of Paediatrics, University of Melbourne, Melbourne, Victoria, Australia; 8 Department of Genetics, Evolution and Environment (GEE), University College London, London, United Kingdom; 9 UCL Great Ormond Street Institute of Child Health (ICH), London, United Kingdom; 10 Department of Neurology, Great Ormond Street Hospital, London, United Kingdom; 11 Victorian Clinical Genetics Services, Melbourne, Victoria, Australia; 12 Division of Biosciences, University College London, London, United Kingdom; 13 Developmental Neurosciences, UCL Great Ormond Street Institute of Child Health, London, United Kingdom; 14 Department of Medicine, University of Melbourne, Melbourne, Victoria, Australia; 15 Austin Health, and Florey Institute of Neuroscience and Mental Health, Melbourne, Victoria, Australia; 16 Department of Pediatrics, Harvard Medical School, Boston, Massachusetts; 17 F.M. Kirby Neurobiology Center, Boston Children’s Hospital, Boston, Massachusetts; 18 Division of Neurology, Hospital for Sick Children, Toronto, Ontario, Canada; 19 Neuroscience and Mental Health Program, SickKids Research Institute, Toronto, Ontario, Canada; 20 Department of Neurology, Boston Children’s Hospital, Boston, Massachusetts; 21 Department of Neurology, Harvard Medical School, Boston, Massachusetts; 22 Children’s Rare Disease Collaborative, Boston Children’s Hospital, Boston, Massachusetts; 23 Department of Neurology, Royal Children’s Hospital, Melbourne, Victoria, Australia; 24 Division of Clinical & Metabolic Genetics, Hospital for Sick Children, Toronto, Ontario, Canada; 25 Department of Paediatrics, Temerty Faculty of Medicine, University of Toronto, Toronto, Ontario, Canada; 26 Department of Molecular Genetics, University of Toronto, Toronto, Ontario, Canada; 27 Division of Newborn Medicine, Department of Pediatrics, Boston Children’s Hospital, Boston, Massachusetts

## Abstract

This study evaluates the proportion of established antisense oligonucleotides (ASO) assessment guidelines that are amenable to ASO therapy approaches for genetically diagnosed infants.

Infantile-onset epilepsies, which affect approximately 1 in 1200 infants, are associated with substantial morbidity and mortality.^1^ Most have presumed genetic etiologies, and timely genetic diagnosis is increasingly possible. There is an urgent need to bridge precision diagnoses to precision therapies to improve outcomes.^2^ Antisense oligonucleotides (ASOs) are nucleic acid-based therapies that bind RNA and are customizable for genetic conditions or genotypes.^3^ While proof-of-concept exists,^4^ their potential utility across genetic epilepsies is unknown. Gene-STEPS is an international multicenter study of rapid genome sequencing (GS) in infantile epilepsies.^5^ We applied established ASO assessment guidelines to genetically diagnosed infants in Gene-STEPS to determine the proportion amenable to ASO therapy approaches.^3,6^

## Methods

This cohort study was approved by the institutional review boards of participating sites and reported per the STROBE guideline. Parents provided written informed consent. From September 2021 to March 2025, we enrolled infants with new-onset unexplained epilepsy or complex febrile seizures and performed rapid GS.^5^ We assessed 160 genetically diagnosed infants (40 per site). Variants were independently classified by 2 assessors using the N = 1 Collaborative (N1C) Variant Assessments Towards Eligibility for Antisense Oligonucleotide Treatment (VARIANT) Guidelines^3^ and Kim and colleagues^6^ splice-switching framework, with discrepancies resolved by multisite review (eFigure in Supplement 1). The primary outcome was the percentage of infants amenable to ASO therapies based on variant assessment (“eligible” or “likely eligible” by N1C guidelines or “probably” or “possibly” amenable by splice-switching framework). The secondary outcome was the percentage of aforementioned infants who could be considered for ASO therapies currently or at seizure onset, incorporating general disease factors and patient-specific phenotypes (eMethods in Supplement 1).

## Results

We assessed 160 infants with genetic epilepsies (74 [46%] female, 86 [54%] male; 172 total variants [152 unique]) for amenability to ASO therapies (Table). N1C guidelines classified 15 of 152 variants (10%) as eligible for various ASO therapy approaches, 7 (5%) likely eligible, 12 (8%) unlikely eligible, 62 (41%) not eligible, and 56 (37%) unable to assess. Splice-switching framework classified 1 variant as probably and 2 possibly amenable to splice correction ASO therapies (1 likely eligible for splice correction, 1 unlikely eligible, 1 not eligible by N1C guidelines). Overall, 24 unique variants from 25 infants (16%) were amenable to ASO therapies: 13 (54%) for knockdown, 7 (29%) wild-type upregulation, 3 (13%) splice correction, and 1 (4%) exon-skipping (Figure). Considering disease and phenotype, 17 of 25 infants (68%) could be currently considered for ASO therapies. An additional 3 (12%), now deceased, could have been considered at seizure onset. Of the remaining 5, 3 have mild disease severity (well-controlled seizures and/or typical development), 1 has a brain malformation, and 1 could be considered if severity worsens. Among 62 variants classified as not eligible, 22 (35%) were autosomal dominant loss-of-function variants in genes possibly eligible for wild-type upregulation, but lacked sufficient data per N1C guidelines to confidently assess. Among 56 variants classified as unable to assess (79% due to unknown variant pathomechanism), 16 (29%) were in genes targeted by an existing ASO, and 8 of 17 infants with these variants had phenotypes consistent with the pathomechanism amenable to existing ASOs.

**Table.** Cohort Demographic, Clinical, and Variant Characteristics

Variable	No. (%) (N = 160)
Demographics	
Sex reported at birth	
Female	74 (46)
Male	86 (54)
Parent-reported race and ethnicity	
Asian	42 (26)
Black	8 (5)
Middle Eastern	9 (6)
White	85 (53)
Multiple	9 (6)
Other^a^	7 (4)
Clinical features	
Seizure onset	
Neonatal (<44 wk postmenstrual age)	39 (24)
Infantile	121 (76)
Epilepsy syndrome at seizure onset	
DEEs	79 (49)
Self-limited epilepsies	47 (30)
Other	34 (21)
Development (at time of variant assessment)^b^	
Developmental delay	93 (64)
Normal development	52 (36)
Deceased (at time of variant assessment)	
Deceased	15 (9)
Living	145 (91)
MCD (at time of variant assessment)	
Yes	21 (13)
No	139 (87)
Type of genome sequencing	
Trio	151 (94)
Duo	8 (5)
Singleton	1 (1)
Variant features (n = 152 unique)	
Zygosity	
Heterozygous	109 (72)
Compound heterozygous	20 (13) (10 pairs)
Homozygous	18 (12)
Hemizygous	3 (2)
Mosaic	2 (1)
Variant type	
Missense	74 (49)
Frameshift	23 (15)
Nonsense	26 (17)
Intronic SNV	8 (5)
In-frame small indel	2 (1)
CNV	17 (12)
Repeat expansion	2 (1)

Abbreviations: ASO, antisense oligonucleotide; CNV, copy number variant; DEEs, developmental and epileptic encephalopathies; MCD, malformation of cortical development; SNV, single-nucleotide variant.

^a^ Other included 7 participants with unknown race, of which 5 reported Hispanic or Latino ethnicity.

^b^ Developmental delay was assessed based on electronic medical records; 15 participants were deceased at the time of ASO assessment and therefore development was not assessed at this time point.

**Figure.**
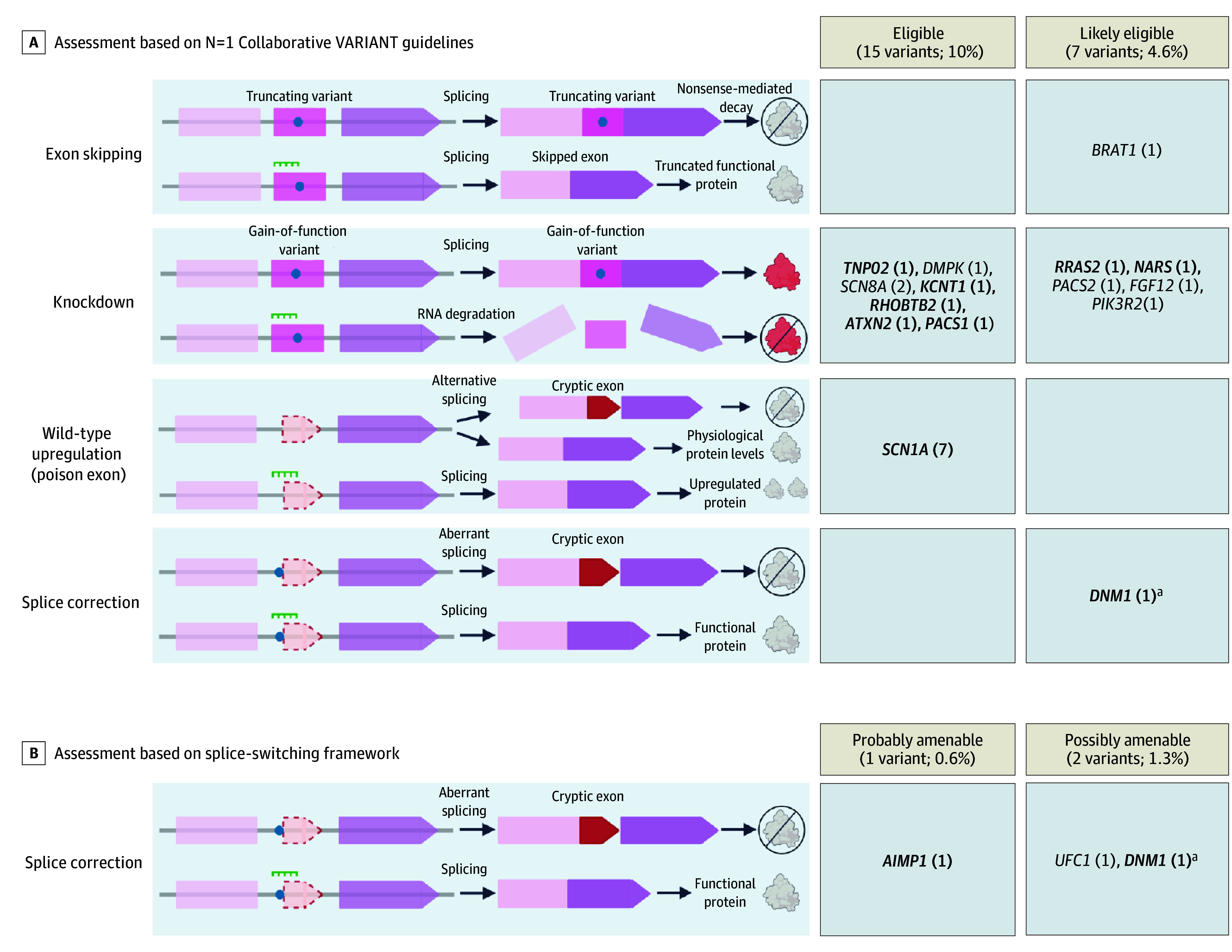
Summary of Antisense Oligonucleotide (ASO) Assessment Results Summary of results for cases with amenable variants based on the (1) N1C Variant Assessments Towards Eligibility for Antisense Oligonucleotide Treatment (VARIANT) guidelines and (2) splice-switching framework. The number of variants identified per gene is indicated in parentheses. Genes in bold indicate that we would consider ASO intervention based on the patient’s current clinical status. The *KCNT1* variant amenable to knockdown was present in 2 patients. ^a^Variant was “likely eligible” under the N1C VARIANT guidelines and also “possibly amenable” under the splice-switching framework.

## Discussion

There is an urgent need for timely precision therapies to optimize outcomes for rare genetic diseases. In our infantile epilepsy cohort, 16% had variants amenable to ASO therapies, and most could be considered currently (68%) or at onset (12%) for therapy development. Our prospectively recruited, rigorously phenotyped cohort provides disease-specific insight into the potential of ASO therapies. A limitation of this study is the 56 variants that were classified as unable to assess due to lack of functional evidence validating variant pathomechanism. Rapid GS in parallel with rapid variant assessment and mechanism validation are needed to identify precision therapy candidates. Implementing this diagnosis-to-therapy framework will require multidisciplinary partnership between clinicians, scientists, and patients.

## Notes

Supplement 1.
eMethods.
**eFigure.** Study workflow
eReferences.

Supplement 2. **Nonauthor Collaborators.** Gene-STEPS Study Group

Supplement 3.
Data Sharing Statement

## References

[1] Zuberi SM, Wirrell E, Yozawitz E, . ILAE classification and definition of epilepsy syndromes with onset in neonates and infants: position statement by the ILAE Task Force on Nosology and Definitions. Epilepsia. 2022;63(6):1349-1397. doi:10.1111/epi.17239 35503712

[2] Howell KB, White SM, McTague A, . International Precision Child Health Partnership (IPCHiP): an initiative to accelerate discovery and improve outcomes in rare pediatric disease. NPJ Genom Med. 2025;10(1):13. doi:10.1038/s41525-025-00474-8 40016282 PMC11868529

[3] Cheerie D, Meserve MM, Beijer D, ; N=1 Collaborative. Consensus guidelines for assessing eligibility of pathogenic DNA variants for antisense oligonucleotide treatments. Am J Hum Genet. 2025;112(5):975-983. doi:10.1016/j.ajhg.2025.02.017 40139194 PMC12120168

[4] Kim J, Hu C, Moufawad El Achkar C, . Patient-customized oligonucleotide therapy for a rare genetic disease. N Engl J Med. 2019;381(17):1644-1652. doi:10.1056/NEJMoa1813279 31597037 PMC6961983

[5] D’Gama AM, Mulhern S, Sheidley BR, ; Gene-STEPS Study Group; IPCHiP Executive Committee. Evaluation of the feasibility, diagnostic yield, and clinical utility of rapid genome sequencing in infantile epilepsy (Gene-STEPS): an international, multicentre, pilot cohort study. Lancet Neurol. 2023;22(9):812-825. doi:10.1016/S1474-4422(23)00246-6 37596007 PMC11860300

[6] Kim J, Woo S, de Gusmao CM, . A framework for individualized splice-switching oligonucleotide therapy. Nature. 2023;619(7971):828-836. doi:10.1038/s41586-023-06277-0 37438524 PMC10371869

