# Supplementary material for: Precision Antisense Oligonucleotide Therapy Amenability for Infantile Genetic Epilepsies
Source: JAMA Neurol. 2026 May 4;83(6):598–601. doi: 10.1001/jamaneurol.2026.1021 (PMC13140085; doi:10.1001/jamaneurol.2026.1021)
Supplement: Supplement 2. — Nonauthor Collaborators. Gene-STEPS Study Group [file jamaneurol-e261021-s002.pdf]

Supplemental Online Content: Nonauthor Collaborators
*First name, last name, and suffix (if applicable) are required and will appear in PubMed.

*Group Name(s): Gene-STEPS Study Group							
***First Name and Middle Initial(s)**	***Last Name**	***Suffix (eg, Jr, III)**	Academic Degrees	Institution	Location (city, state/province, country)	Role or Contribution, eg, chair, principal investigator	Group (if more than 1 Group listed in the byline) and/or Subgroup (eg, Steering Committee)
Joanna	Cobb		PhD	Murdoch Children's Research Institute			
Anna J S	Griffiths		BSc	Murdoch Children's Research Institute			
Edward J	Higgenbotham		MSc	Hospital for Sick Children			
Puneet	Jain		MD, DM	Hospital for Sick Children			
Nicole S Y	Liang		MSc, CGC	Hospital for Sick Children			
Sebastian	Lunke		PhD	Murdoch Children's Research Institute			
Christian R	Marshall		PhD	Hospital for Sick Children			
Catherine	Marx		PhD	Hospital for Sick Children			
Lyndsey	McRae		BA, RN(EC), MN, NP-PHC	Hospital for Sick Children			
Jimmy N H	Nguyen		BSc	Hospital for Sick Children			
Wanqing	Shao		PhD	Boston Children's Hospital			
Beth R	Sheidley		MS, CGC	Boston Children's Hospital			
Lacey	Smith		MS, CGC	Boston Children's Hospital			
Zornitza	Stark		BM BCh, DM	Murdoch Children's Research Institute			
Susan M	White		MBBS	Murdoch Children's Research Institute			
