# Supplementary material for: Precision Antisense Oligonucleotide Therapy Amenability for Infantile Genetic Epilepsies
Source: JAMA Neurol. 2026 May 4;83(6):598–601. doi: 10.1001/jamaneurol.2026.1021 (PMC13140085; doi:10.1001/jamaneurol.2026.1021)
Supplement: Supplement 3. — Data Sharing Statement [file jamaneurol-e261021-s003.pdf]

Data Sharing Statement

Sherrill. Precision Antisense Oligonucleotide Therapy Amenability for Infantile Genetic Epilepsies. *JAMA Neurol*. Published May 04, 2026. doi:10.1001/jamaneurol.2026.1021

Data
Data available: Yes
Data types: Deidentified participant data
How to access data: Deidentified demographic, clinical, variant, and assessment data is available at www.dgamalab.org/resources. Reported variants were deposited into public databases (e.g., ClinVar) per the policies of the clinically accredited laboratories who reported the variants.
When available: With publication

Supporting Documents
Document types: None

Additional Information
Who can access the data: Deidentified demographic, clinical, variant, and assessment data is available at www.dgamalab.org/resources. Reported variants were deposited into public databases (e.g., ClinVar) per the policies of the clinically accredited laboratories who reported the variants.
Types of analyses: Deidentified demographic, clinical, variant, and assessment data is available at www.dgamalab.org/resources. Reported variants were deposited into public databases (e.g., ClinVar) per the policies of the clinically accredited laboratories who reported the variants.
Mechanisms of data availability: Deidentified demographic, clinical, variant, and assessment data is available at www.dgamalab.org/resources. Reported variants were deposited into public databases (e.g., ClinVar) per the policies of the clinically accredited laboratories who reported the variants.
